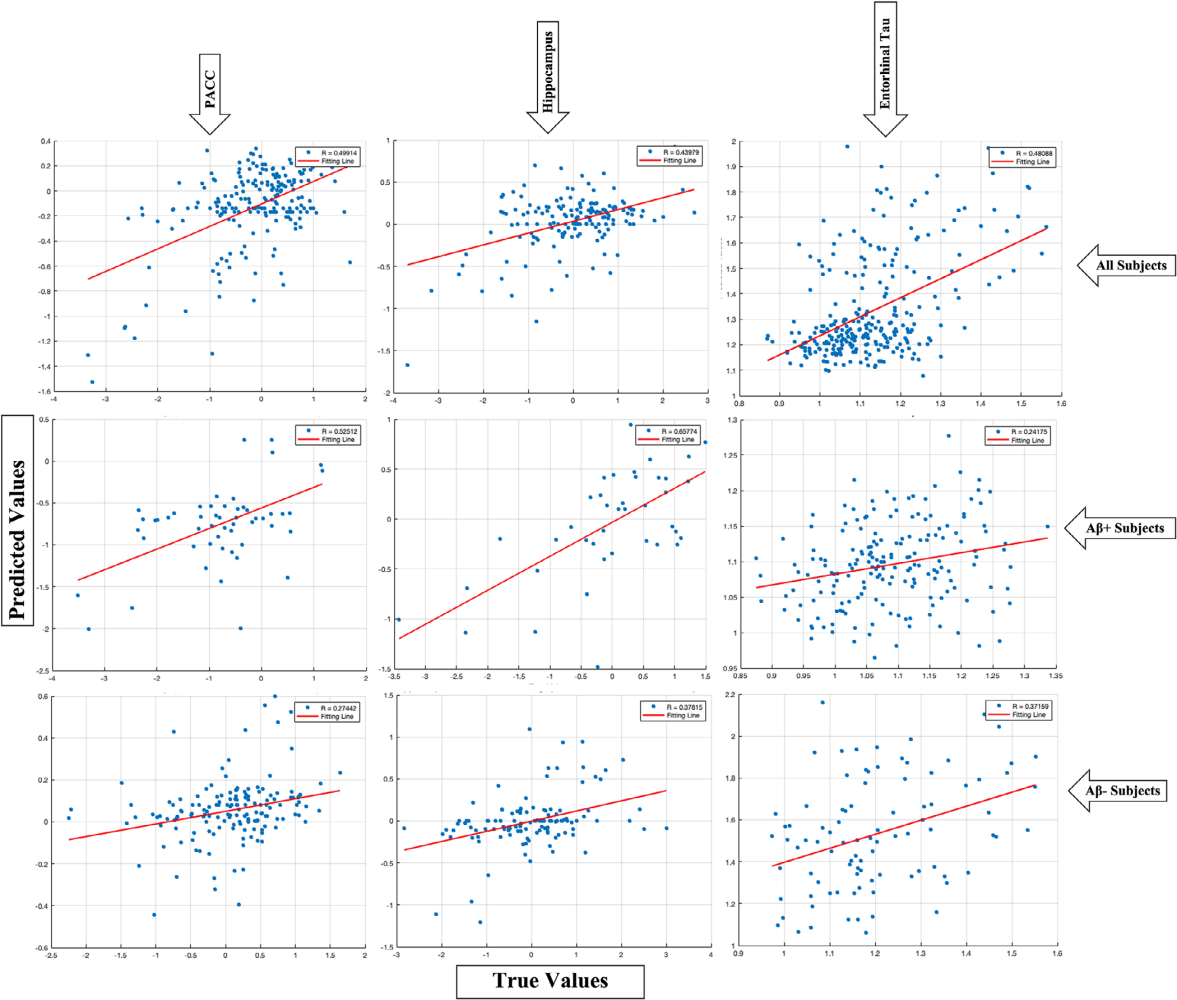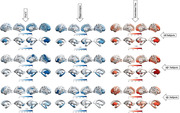# Deciphering the Synergy Between Amyloid‐Beta and Tau Pathologies in Preclinical Alzheimer's Disease

**DOI:** 10.1002/alz.092180

**Published:** 2025-01-09

**Authors:** Jafar Zamani, Amirali Vahid, Elveda Gozdas, Hadi Hosseini, C‐Brain Lab, Department of Psychiatry and Behavioral Sciences, School of Medicine, Stanford University, Stanford, CA, 94304, USA

**Affiliations:** ^1^ Stanford University, Stanford, CA USA

## Abstract

**Background:**

The extracellular deposition of amyloid‐beta (Aβ) and intracellular aggregation of hyperphosphorylated tau proteins stand as histopathological hallmarks of Alzheimer's disease (AD). The spatial‐temporal relationship between the Aβ pathway and tau pathophysiology in AD, plays a crucial role in comprehending the pathogenesis and progression of AD. Understanding the interrelationship between Aβ and tau, is crucial to elucidate the failure of previous therapeutic strategies and understanding AD progression and will inform the next generation of clinical trials. We employed a robust machine learning approach to predict entorhinal tau levels, using Aβ SUVR‐identified pattern. Subsequently, we employed the identified Aβ SUVR pattern to predict long‐term hippocampal volume and Preclinical Alzheimer Cognitive Composite (PACC) cognitive measurement at five‐year follow‐up, as indicators of neurodegeneration and cognitive decline, respectively.

**Method:**

We analyzed PET measures for amyloid and tau in a cohort of 509 participants, 233 participants (104 Aβ+, 129 Aβ−) from ADNI as training data and 276 participants (93 Aβ+, 183 Aβ−) from Harvard Aging Brain Study (HABS) as an independent test set. We employed support vector regression (SVR) as a machine learning regression method for predicting entorhinal tau levels based on Aβ SUVR pattern. Feature‐selection and cross validation were performed to build and train the model using ADNI training data and the performance of the model was tested using independent HABS data. We also tested if the trained SVR model can successfully predict hippocampal volume and PACC scores at 5‐year follow‐up using baseline Aβ SUVR pattern. We constructed separate predictive models for Aβ+ and Aβ‐ subjects.

**Result:**

The SVR model trained on ADNI Aβ data successfully predicted entorhinal tau, future hippocampal volume and PACC scores in an independent HABS dataset with a correlation accuracy of 0.50, 0.68 and 0.55, respectively (Figure 1). Particularly, Aβ load in the medial orbitofrontal and rostral middle frontal regions contributed the most to predicting entorhinal tau and future hippocampal volume and cognitive outcomes (Figure 2).

**Conclusion:**

Our study indicates that amyloid load in medial prefrontal regions may signal subsequent tau accumulation in the entorhinal cortex, and future neurodegeneration and cognitive decline, suggesting its potential role as a marker for AD progression.